# Dynamic regulation of integrin activation by intracellular and extracellular signals controls oligodendrocyte morphology

**DOI:** 10.1186/1741-7007-3-25

**Published:** 2005-11-12

**Authors:** Inger Marie Olsen, Charles ffrench-Constant

**Affiliations:** 1 Departments of Pathology and Medical Genetics, University of Cambridge, Tennis Court Road, Cambridge CB2 1QP, UK; 2 Centre for Basic Psychiatric Research, Aarhus University Hospital, Skovagervej 2, DK-8240 Risskov, Denmark

## Abstract

**Background:**

Myelination requires precise control of oligodendrocyte morphology and myelin generation at each of the axons contacted by an individual cell. This control must involve the integration of extracellular cues, such as those on the axon surface, with intrinsic developmental programmes. We asked whether integrins represent one class of oligodendrocyte cell-surface receptors able to provide this integration.

**Results:**

Integrins signal via a process of activation, a conformational change that can be induced either by "outside-in" signals comprising physiological extracellular matrix ligands (mimicked by the pharmacological use of the divalent cation manganese) or "inside-out" signalling molecules such as R-Ras. Increasing levels of outside-in signalling via the laminin receptor α6β1 integrin were found to promote oligodendrocyte processing and myelin sheet formation in culture. Similar results were obtained when inside-out signalling was increased by the expression of a constitutively-active R-Ras. Inhibiting inside-out signalling by using dominant-negative R-Ras reduces processes and myelin sheets; importantly, this can be partially rescued by the co-stimulation of outside-in signalling using manganese.

**Conclusion:**

The balance of the equilibrium between active and inactive integrins regulates oligodendrocyte morphology, which is itself regulated by extrinsic and intrinsic cues so providing a mechanism of signal integration. As laminins capable of providing outside-in signals are present on axons at the time of myelination, a mechanism exists by which morphology and myelin generation might be regulated independently in each oligodendrocyte process.

## Background

The process of myelination in the CNS requires a remarkable morphological transformation by newly-formed oligodendrocytes, with processes contacting and extending along each axon before elaborating a myelin membrane to enwrap the axon multiple times to create a sheath. This differentiation step is tightly controlled, as indicated by the formation of processes each with sufficient membrane for a sheath thickness that has a precise relationship with final axon diameter [1]. In order to ensure that the precise amount of myelin is formed at the right developmental stage and in the correct place, a key component of oligodendrocyte behaviour during myelin formation must be the integration of multiple extrinsic signals at the axon surface along with intrinsic programmes, such as autonomous developmental timers of differentiation. These points of integration are therefore important for our understanding of myelination and may facilitate the development of strategies to promote remyelination.

**Figure 1:**
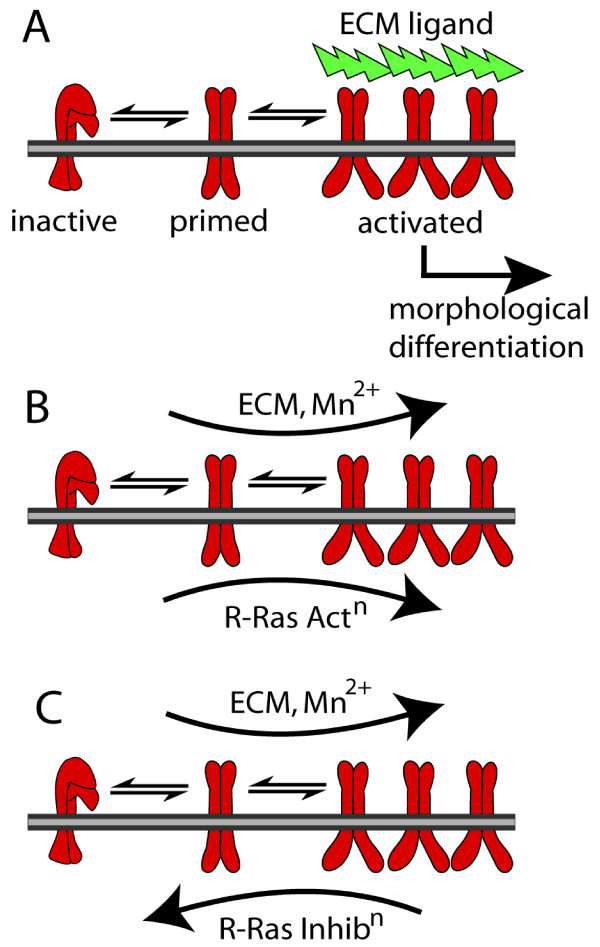
Strategies for manipulating integrin activation in oligodendrocytes. Panel A shows the equilibrium between 3 different conformations of integrin; inactive, primed and activated. Only the latter assembles a signalling complex and promotes morphological differentiation of oligodendrocytes, as manifested by complex processes and the formation of myelin sheets. Panel B shows the 2 methods used in this study to promote activation; outside-in signalling using high extracellular matrix (ECM) ligand concentrations or the divalent cation Mn^2+ ^(results in Fig 2), and inside-out signalling using active R-Ras (Fig 3). Panel C illustrates the logic of the experiment shown in Fig 4 to confirm that integrins integrate these extracellular and intracellular cues, showing how outside-in signalling would be predicted to overcome the effects of R-Ras inhibition on oligodendrocyte morphology and myelin sheet formation.

One important group of candidate integrative molecules are the integrins, the cell surface receptors of extracellular matrix proteins. Integrins comprise two transmembrane chains, termed α and β, with a ligand-binding site formed by the head domain of the two chains [2]. Recent work has established that integrins exist in at least three different confirmations on the cell surface, each in a dynamic equilibrium with one another (Fig 1A) [3-7]. Inactive integrins are folded over, have a low binding affinity for ligand and do not signal. Primed integrins are straightened, and bind ligand with higher affinity as a result of shape changes within the head domain. Activated integrins have bound ligand leading to receptor clustering, and have undergone a further shape change in the β chain leading to separation of the two cytoplasmic domains, thereby allowing formation of the signalling complex (termed "outside-in" signalling). Since the change of shape can be transmitted across the membrane in either direction, activation can also be achieved by so called "inside-out" signals. These separate cytoplasmic domains and induce changes in the extracellular ligand-binding site that increase receptor affinity, leading to ligand binding, integrin clustering and signalling. As a result, integrin activation and formation of the signalling complex is regulated by the integration of both extrinsic ligand concentrations and the activity of (intrinsic) 'inside out' signalling pathways.

Oligodendrocytes express 5 integrins; αVβ1, αVβ3, αVβ5 and αVβ8 as well as α6β1 [8,9]. In cell culture, the αV integrins promote proliferation and migration [10,11], while the laminin receptor, α6β1, promotes differentiation (as measured by myelin sheet formation) and survival [12,13]. In vivo, mice lacking α6 show increased apoptosis of newly-formed oligodendrocytes [14]. Transplantation of cells expressing dominant-negative β1 integrins into focal demyelinated lesions in the adult rat reduced the extent of remyelination [15]. We therefore focused on α6β1 to examine the hypothesis that integrin activation provides a mechanism for the integration of extrinsic and intrinsic signals in oligodendrocyte differentiation. Our goal was to test two key predictions of the hypothesis (Fig 1B and 1C): (i) that either extracellular or intracellular signals regulating integrin activation can control oligodendrocyte morphology, and (ii), that the effects of inhibition of one class of signals could be partially overcome by stimulation of the other, so demonstrating integration of signals by the integrin.

## Results

**Figure 2:**
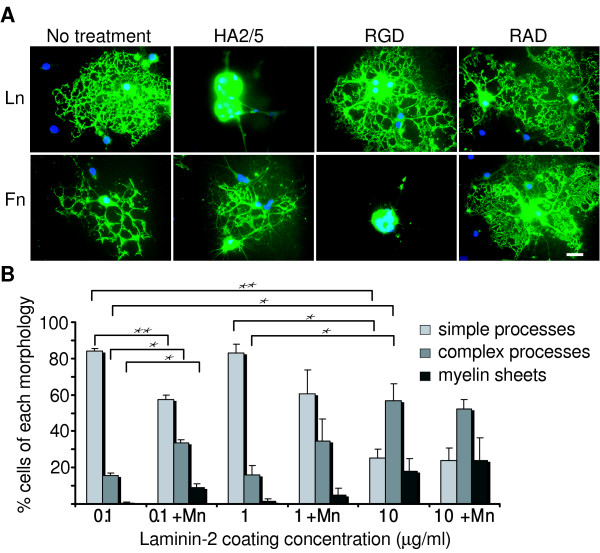
Outside-in integrin signalling in oligodendrocytes. Panel A shows that morphological differentiation of oligodendrocytes on laminin-2 requires integrins. A monoclonal anti-β1 integrin-blocking antibody (Ha2/5) inhibits oligodendrocyte process formation on laminin-2 (Ln) but not fibronectin (Fn). In contrast, RGD peptides that block αV integrins inhibit the morphological differentiation on Fn. No change is seen with control RAD peptides that do not perturb integrin function. Panel B shows that integrin activation by outside-in signalling promotes differentiation. Increasing laminin-2 concentrations are associated with greater process complexity and myelin sheet formation, with significant increases seen between 1 and 10 μg/ml. Mn^2+ ^(Mn) also promotes differentiation, with significant effects seen at the lowest laminin-2 concentration (0.1 μg/ml). Note that the addition of Mn^2+ ^has no significant effect on morphology at the higher laminin concentrations. Single and double asterix indicate significance levels of p < 0.05 and p < 0.01 respectively, calculated using Student's paired t-test. Scale bar in panel A is 20 μm.

We have shown previously that laminin-2 substrates promote the formation of a differentiated morphological phenotype in oligodendrocytes, as evidenced by increased elaboration of processes and myelin sheets, and that α6 and β1 integrin subunits are found throughout the process network of oligodendrocytes [12]. To confirm that the change in phenotype was α6β1 integrin dependent (and can therefore be used as an assay of integration of inside-out and outside-in signalling), oligodendrocyte morphology was analyzed in the presence of the monoclonal antibody Ha2/5 (that blocks β1 integrins) and RGD peptides (that mimic the peptide recognition sequence within the extracellular matrix molecules recognised by αV integrins and so block all the oligodendrocyte αV integrins [16]). While confirming previous results with polyclonal anti-β1 antibody, we also found that the monoclonal Ha2/5 antibody blocked morphological differentiation on laminin-2 (Fig 2A). In contrast, this antibody had little effect on fibronectin substrates, which promote process outgrowth but not myelin sheet formation and on which the RGD peptide was an effective inhibitor (Fig 2A). Since αVβ1 and α6β1 are the only β1 integrins expressed on oligodendrocytes, and because RGD peptides would be expected to block αVβ1, we conclude that morphological differentiation leading to myelin sheet formation by oligodendrocytes on laminin-2 is α6β1-dependent.

To confirm that extracellular signals regulate oligodendrocyte morphology by activation of α6β1, oligodendrocyte precursor cells were plated on increasing concentrations of laminin-2 and allowed to differentiate into oligodendrocytes. Oligodendrocyte morphology was scored as having either simple (primary) processes only, complex (secondary and tertiary) processes or myelin sheets, reflecting the different changes associated with axon contact and sheath formation. Increasing the laminin-2 coating concentration from 1 to 10 μg/ml resulted in a significantly greater degree of morphological differentiation (Fig 2B). To exclude the possibility that this represented signalling by a second non-integrin receptor rather than α6β1 integrin, we also examined the effect of adding Mn^2+ ^to the tissue culture media. This divalent cation replaces the Ca^2+ ^normally present in the integrin ligand-binding domain and activates the integrin as a result of the conformational changes associated with the substitution. At low coating concentrations of laminin-2 (0.1 μg/ml), Mn^2+ ^significantly increased morphological differentiation, but had no effect at higher concentrations (Fig 2B). This result would be expected if either high ECM concentrations (1 μg/ml and above) or Mn^2+ ^were sufficient to activate the oligodendrocyte α6β1 integrins effectively, while lower ECM concentrations only elicited partial activation. Consequently, Mn^2+ ^promotes further differentiation at low ECM concentrations (by activating those integrins not already activated by the ECM) but has no significant additive effect at the high ECM concentrations.

**Figure 3:**
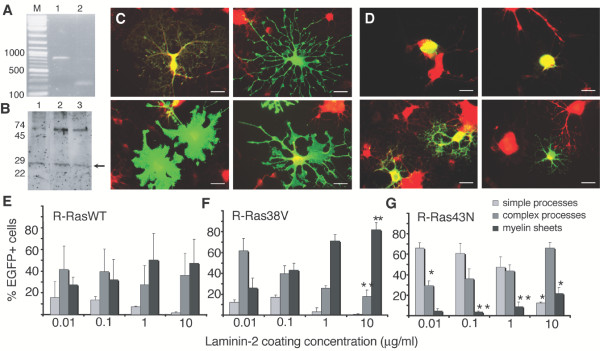
Inside-out integrin signalling in oligodendrocytes. Panel A shows an RT-PCR experiment on cDNA obtained from cultured oligodendrocytes, grown as described in the Methods. Lane 1 – size markers (nt); lane 2 – ADAM9 (control); lane 3 – R-Ras. Panel B shows a western blot of lysates from 3 different sets of cultured oligodendrocytes (lanes 1–3) using anti-R-Ras antibodies. All 3 lanes show a band at the predicted size (23 kD) of R-Ras (arrow). Size markers (kDa) shown on the left. These 2 experiments therefore confirm that oligodendrocytes express R-Ras. Panel C shows the effect of expressing constitutively active R-Ras (R-Ras38V) in oligodendrocytes, while Panel D shows the effect of expressing the dominant-negative R-Ras43N; all 4 micrographs in each panel show representative examples of transfected cells visualised by antibodies against EGFP (green) and CNPase (red). Note the different morphologies observed, with elaborate processes and large sheets seen in some cells expressing R-Ras38V and stunted processes in some expressing R-Ras43N. The effects of R-Ras38V and R-Ras43N at different laminin-2 concentrations are quantified in Panels F and G, with control cells (expressing wild-type R-Ras) in these experiments shown in panel E. These show the increased complexity of R-Ras38V cells at all laminin-2 concentrations when compared with wild-type cells, in contrast to the decreased complexity of the R-Ras43N cells. Single and double asterix indicate significance levels of p < 0.05 and p < 0.01 respectively, calculated using Student's paired t-test. Scale bars in panels C and D are 20 μm.

The effect of intracellular signalling pathways that regulate integrin activation was explored. Both RT-PCR and western blotting studies confirmed the presence of R-Ras in oligodendroglial cells (Fig 3A,B), shown to regulate integrin activation in other cell types [17,18]. To manipulate R-Ras signalling, transient transfections of primary oligodendrocyte precursor cell populations were used to express either wild-type (control) or mutant forms of R-Ras [19]. Expression of a constitutively active mutant form of R-Ras (38V) resulted in a dramatic increase in sheet formation by the oligodendrocytes (Fig 3C). Quantification revealed that this increase was significantly greater than that seen in cells expressing wild-type R-Ras at the highest laminin-2 concentrations tested, with >80% of the cells expressing sheets at 10 μg/ml (Fig 3F).

Conversely, a dominant negative R-Ras construct (43N) reduced the degree of morphological differentiation seen in response to laminin-2 (Fig 3D). When compared with cells expressing wild-type R-Ras, the percentage of oligodendrocytes with a complex morphology was significantly reduced at laminin-2 concentrations above 0.1 μg/ml, with the percentage exhibiting a simple morphology correspondingly increased (Fig 3G). Together with the data using the constitutively-active R-Ras38V, these results point to the presence on the surface of the oligodendrocyte of a dynamic equilibrium between inactive and activated α6β1 that can be displaced in either direction by manipulation of R-Ras signalling. Moreover, when considered with the experiments using high laminin-2 coating concentrations and/or Mn^2+^, they confirm our first prediction that either intracellular or extracellular cues regulating activation can control oligodendrocyte morphology.

**Figure 4:**
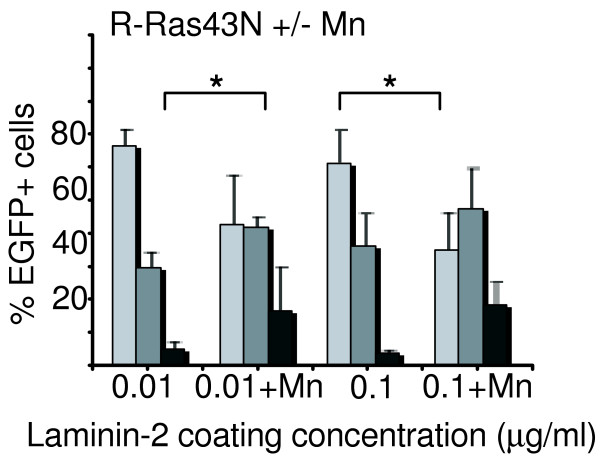
Outside-in integrin signalling rescues the effect of dominant-negative R-Ras43N. In the same set of experiments illustrated in Fig 3, Panel G, addition of Mn^2+ ^significantly increases oligodendrocyte complexity in the cells expressing dominant-negative R-Ras. *p < 0.05.

To test the second prediction of the hypothesis that this equilibrium provides a mechanism for integrating intracellular and extracellular cues, we determined whether the effects of inhibiting activation by dominant negative R-Ras (an "inside-out" signal) could be overcome by stimulating activation using Mn^2+ ^(an "outside-in" signal), as illustrated in Fig 1C. Mn^2+ ^increased the extent of sheet formation, and decreased the percentage of cells with a simple morphology, in cells expressing the dominant negative R-Ras construct at the two lower laminin concentrations tested (Fig 4). Because Mn^2+ ^acts directly on the integrin ligand binding domain, we can conclude that the increase in sheet formation reflects a direct effect on the integrin and therefore on the equilibrium between inactive and active integrins, rather than via modulation of separate downstream signalling pathways.

## Discussion

These results show that the equilibrium between active and inactive integrins regulates oligodendrocyte morphology and myelin sheet formation. The balance of this equilibrium is determined by a combination of intracellular signals and extracellular ligand concentrations, with the two classes of signal integrated by individual integrin heterodimers. Manipulation of the equilibrium provides one mechanism by which both the number of myelin sheaths formed by an individual oligodendrocyte, as well as their thickness, could be regulated during CNS development.

To be relevant to myelination in vivo, our results require the presence of signals in the developing brain that can promote both outside-in and inside-out integrin signalling. We have shown previously that laminin α2 (the α chain present in the laminin trimers 2,4 and 12) is present on the surface of axons in embryonic brainstem at the time of myelination [14], extending a previous report of laminin α2 expression by Purkinje cells in the myelinating postnatal cerebellum [20]. Binding of this laminin would provide an outside-in signal, as shown in the experiments using increasing laminin concentrations. A number of different extracellular signals might trigger the intracellular pathways that promote inside-out signalling. For example, our previous work has defined a role for growth factors and their receptors in integrin activation via "inside-out" signalling. In studies on cell proliferation of oligodendrocyte precursors, αvβ3 integrin was activated by PDGF [21]. Activation of αvβ3 had been previously described in response to VEGF in endothelial cells [22], suggesting this might represent a general mechanism for integrin/growth factor interactions. Two other extracellular signalling systems, eph/ephrins and semaphorins, have also been shown to alter integrin activation [23-25]. Equally, the key intracellular components of the inside-out signalling pathway are present in oligodendrocytes. We have reported here that R-Ras is expressed in oligodendroglia. Recent work has shown that the interaction of the cytoskeletal protein talin with the β integrin cytoplasmic domain represents a final common pathway for the signals that promote activation [26], and we have also found that talin is expressed in oligodendroglial cells (Uli Forster and C ff-C, unpublished observations) consistent with such a role in these cells.

These observations show that there are multiple potential signalling pathways that might regulate the equilibrium of active and inactive integrins. Together with our present results, they support a model by which this equilibrium provides a dynamic means of integrating multiple signals to control the timing of myelination and the extent of myelin membrane. The model is based on the assumption that, under physiological conditions during normal development where growth factors are present in limiting concentrations, two classes of signals are required to generate sufficient numbers of activated integrins. So, for example, the laminin on the axon surface would be necessary for integrin signalling and myelination, but may not be sufficient without a further contribution to integrin activation from intracellular ("inside-out") signals. These "inside-out" signals could be generated by growth factor signalling, or alternatively from intracellular pathways driven by autonomous developmental clocks [27], and would then effectively act as timers of myelination. Equally, while these intracellular signals may initiate the myelination process, the amount of myelin formed (and hence the number of wraps) would then be determined by the concentration of extracellular ligand on the axon surface, with higher concentrations increasing integrin activation and so the amount of myelin produced. A precedent for wrap-number being determined by the concentration of axon-surface molecules is provided by the PNS, where transmembrane isoforms of neuregulin regulate Schwann cell myelination [28,29]. There is no evidence that laminins provide quantitative signals to the myelinating oligodendrocytes, but the observation that laminin-2 deficient dy/dy mutant mice show an increased G ratio (reflecting thinner myelin) in the CNS [30] would be consistent with such signals, and further studies are required.

## Conclusion

Oligodendrocyte morphology is regulated at least in part by the balance of active and inactive integrins (Fig 1A). Such a model provides important insights into two aspects of oligodendrocyte biology. First, a single oligodendrocyte can myelinate multiple axons, and the thickness of each sheath (and therefore the amount of myelin membrane produced at the end of each process) is independently regulated by the axon it ensheaths. Clearly, this remarkable example of local control of cell-cell interactions cannot be regulated entirely by changes in transcription or translation initiated in the cell body. However, the control of integrin signalling by alterations of the equilibrium between active and inactive integrins provides a plausible mechanism by which integrin ligands on the axon could regulate membrane formation and morphological changes independently within each process. Second, one hypothesis for the failure of remyelination in MS (the dysregulation hypothesis) is that the loss of the coordination between promyelinogenic signals, rather than the loss of any one specific factor, prevents repair [31]. The possibility that integrative signalling molecules such as integrins might provide targets for drug therapies designed to promote remyelination by activation and promotion of signalling in the absence of the usual upstream cues therefore requires further investigation.

## Methods

### Oligodendrocyte cultures

Purified oligodendrocyte precursor cells were obtained from primary cultures of newborn Sprague-Dawley rat forebrains and were isolated by mechanical dissociation and differential adhesion as described previously [8]. The precursor cells were then plated on 8-well Permanox chamber slides (Nunc) pre-coated with fibronectin (10 μg/mL; Sigma) or laminin-2 (0.01, 0.1, 1 and 10 μg/mL; Sigma human placental laminin) at 20,000 cells/well. For both the blocking experiments and the Mn^2+ ^experiments, the cells were allowed to attach to the substrate for 2 h at 37°C before adding either blocking reagents or Mn^2+^. Ha2/5 antibody (PharMingen) was used at 10 μg/mL, blocking peptides (RGD and RAD; Calbiochem) were used at 0.1 μg/mL and Mn^2+ ^(MnCl_2_·4H_2_O; Sigma) was freshly prepared and used at 50 μg/mL. After 3 days of differentiation in SATO medium [8] at 37°C, 7.5% CO_2_, cells were fixed with 4% paraformaldehyde for 15 min at room temperature. The cells were permeabilised and blocked in a one-step procedure with 10% normal goat serum (NGS, Sigma) and 0.1% TritonX-100 (NBS Biologicals) in PBS for one hour at room temperature prior to immunostaining.

### Transfection

After 10–12 days in vitro, the forebrain cultures were rinsed twice with DMEM (Sigma) supplemented with 10% fetal bovine serum (Sigma) and subsequently transfected overnight with the following constructs; pEGFP-C1/R-Ras-wt, pEGFP-C1/R-Ras-38V and pEGFP-C1/R-Ras-43N using FuGene 6.0 (Roche). The R-Ras constructs have been previously described [19], and were a kind gift of Prof Alan Hall (UCL, London, UK). They were recloned into the pEGFP-C1 vector (Clontech) following digestion with BamHI and XbaI. The cells were then washed three times with DMEM (10% fetal bovine serum) and 24 h later they were plated onto laminin-2 substrates as described above at 40,000 cells/well. The cells were differentiated for 3 days before fixation, permeabilisation and blocking in preparation for the immunostaining.

### Immunostaining

Cells were washed three times with PBS and were incubated with appropriate dilutions of the primary antibodies in a buffer of PBS, 1% NGS and 0.1% Triton X-100 for 1–2 h at room temperature or overnight at 4°C. Myelin basic protein (MBP) staining was carried out using a 1:50 dilution of rat anti-MBP (82–87) (Serotec). A 1:100 dilution of mouse anti-2',3'-cyclic nucleotide 3'-phosphohydrolase (CNPase clone 11-5B, Sigma) was used for CNPase staining. For enhanced green fluorescence protein (EGFP) staining rabbit anti-GFP IgG (1 μg/mL corresponding to 1:1000, Molecular Probes) was used. After washing with PBS, the cells were incubated with secondary antibodies (Jackson ImmunoResearch Laboratories INC.) diluted 1:100 in PBS with 1% NGS for 1 h at room temperature.

### RT-PCR and Western blotting

Total RNA was isolated from oligodendrocyte precursor cells and cDNA was made using a first strand cDNA synthesis kit (Amersham) according to the manufacturer's instructions. For the RT-PCR the primer sequences were: ADAM9 (control) GCCTGACGAAGCCTACA and GTCCACGCTTCCTCCTAT; R-Ras GGTTGGGAACAAGGCAGATC and GCAGGACACAGGGACATCC. For western blotting, differentiated oligodendrocytes were lysed and scraped off in 250 μL lysis buffer (50 mM Tris-HCl, 5 mM EDTA, 150 mM NaCl, 1% Triton X-100, pH7.4), with the protease inhibitors phenylmethylsulfonyl fluoride (2 mM), pepstatin (1 μg/mL), leupeptin (5 μg/mL) and aprotinin (2 μg/mL)) per 10 cm tissue culture dish. The cell lysates were incubated for 30 min and centrifuged (13,000 rpm) for 10 min at room temperature. Protein concentrations were determined using a detergent compatible Lowry protein assay (BioRad DcProtein assay). Denatured, boiled proteins were separated by SDS-PAGE (12% pre-cast Tris-HCl minigels; BioRad) and transferred onto a 0.45 μm nitrocellulose membrane (HybondC, Amersham). The membrane was blocked in 4% bovine serum albumin (Sigma) in TBST (TBS with 0.1% Tween 20) for 1 h at room temperature, and further incubated with rabbit anti-R-Ras (BD PharMingen; 1:1000) in blocking buffer overnight at 4°C. After washing with TBST, the membrane was incubated with a horseradish peroxidase-conjucated secondary antibody (Amersham) in 2% blocking buffer for 1 h at room temperature. The membrane was washed and the immunoreactive proteins were detected using enhanced chemiluminescence (Amersham).

### Scoring

MBP+/EGFP+ cells were assigned to one of three morphological categories. Cells that had only primary processes were assigned to the first category, which was denoted 'simple processes'. Cells with secondary and tertiary processes were assigned to a second category denoted 'complex processes'. Cells with myelin sheets (as evidenced by continuous staining filling in between the processes) were assigned to a third category denoted 'myelin sheets'. In each assay, 50 MBP+/EGFP+ oligodendrocytes were scored, with the results being the M ± SEM of 3 independent experiments.

## Authors' contributions

IMO carried out all experimental analyses and drafted the manuscript. C ff-C conceived of the study, participated in its design and coordination and helped to draft the manuscript. Both authors read and approved the final manuscript.
